# How diverse is the diet of adult South Africans?

**DOI:** 10.1186/1475-2891-10-33

**Published:** 2011-04-17

**Authors:** Demetre Labadarios, Nelia Patricia Steyn, Johanna Nel

**Affiliations:** 1 Centre for the Study of Social and Environmental Determinants of Nutrition, Knowledge Systems: Human Sciences Research Council, Cape Town, South Africa (RSA; 2 Department of Logistics, University of Stellenbosch, Stellenbosch, RSA

**Keywords:** Dietary diversity, Adults, Africa, Food groups, Food Security, 24 Hour Recall

## Abstract

**Background:**

The objective of the current study was to measure dietary diversity in South Africans aged 16 years and older from all population groups as a proxy of food security.

**Methods:**

A cross-sectional study representative of adults from all specified ages, provinces, geographic localities, and socio-economic strata in South Africa was used (n = 3287). Trained interviewers visited participants at their homes during the survey. Dietary data was collected by means of a face validated 24 hour recall which was not quantified. A dietary diversity score (DDS) was calculated by counting each of 9 food groups. A DDS <4 was regarded as reflecting poor dietary diversity and poor food security.

**Results:**

The provinces with the highest prevalence of poor dietary diversity (DDS <4) were Limpopo (61.8%) and the Eastern Cape (59.6%). By contrast, only 15.7% of participants in Western Cape had a low score. Participants in tribal areas (63.9%) and informal urban areas (55.7%) were by far the worst affected. There were significant differences in DDS by Living Standards Mean (LSM) analysis (p < 0.05) with the lowest LSM group having the lowest mean DDS (2.93).The most commonly consumed food groups were cereals/roots; meat/fish; dairy and vegetables other than vitamin A rich. Eggs, legumes, and vitamin A rich fruit and vegetables were the least consumed.

**Conclusion:**

Overall the majority of South Africans consumed a diet low in dietary variety. The tribal areas and informal urban areas were worst affected and eggs, legumes and vitamin A rich fruit and vegetables, were the least consumed.

## Introduction

A diet which is sufficiently diverse reflects nutrient adequacy [1]. This statement is based on the fact that there is no single food which contains all required nutrients for optimal health. Hence, the more food groups included in a daily diet the greater the likelihood of meeting nutrient requirements. Monotonous diets, based mainly on starches e.g. maize, bread, have been closely associated with food insecurity [1]. According to Hoddinott [2], dietary diversity is an outcome measure of food security at the individual or household level, while food security is defined as access by all people at all times to enough food for an active, healthy life [3].

Recently there has been a lack of clarity regarding food security and dietary diversity status of the South African population. This being due in part to large temporal differences in food security obtained by different surveys when using the same hunger scale [4,5]. It is difficult to determine whether the higher levels of hunger in 2005 (51.6%) [4] compared with that of 2008 (25.9%) [5] reflect an improvement in food security or whether other factors including differences in the sampling methodologies of the relevant surveys may have influenced the outcomes. This was one of the reasons food diversity was evaluated in 2009 (current study) by the Human Sciences Research Council (HSRC) of South Africa as an additional measure of food security, since one measure of food security is not usually used on its own.

In the national child population (1-9 years) surveyed in 1999 [in the National Food Consumption Survey (NFCS) of South Africa [4], a dietary diversity score (DDS) was determined and validated against both mean adequacy ratio (MAR) of the diet and anthropometric status [6]. MAR and DDS showed significant correlations with weight for age (W/A) and height for age (H/A) Z scores, indicating a strong relationship between DDS and child growth. The NFCS further showed that the prevalence of stunting was highest in tribal areas and in certain provinces, namely Eastern Cape, Northern Cape and Limpopo. The most commonly consumed foods at the time were maize, tea, sugar and bread, indicating a very monotonous diet [7].

The dietary data from the NFCS further indicated that the diet of many children was low in energy and certain essential micronutrients [7]. It was noted that dietary diversity was poor and that this could be assumed to reflect poor food security [1,2]. A subsequent secondary dietary analysis showed that participating children had a mean DDS of 3.6 (SD = 1.4)[6]. Furthermore, a DDS of at least 4 was shown to be the lowest minimum requirement and provided a specificity of 70% and a sensitivity of 75% of at least at 50% MAR of the overall diet.

Determining dietary diversity in South African adults has not been possible to date since there are no national dietary data on adolescents or adults. Hence, the aim of the present study was to include a dietary assessment in 2009 of adolescents and adults (16 years and older) participating in the national South African Social Attitudes Survey (SASAS) and to calculate DDS for this group. Further, to assess the determinants of dietary diversity in this population.

## Methods

The SASAS has been conducted annually by the HSRC since 2003. The aim of the survey is to track longitudinally the public's attitudes, beliefs and behavior patterns on social issues [8].

The survey has been designed to yield a nationally representative sample of people aged 16 years and older. The sampling frame for the survey is enumerator areas adjacent (i.e. including the household next door to the one in the master sample) to the HSRCs Master Sample which was designed in 2006 and consists of 1 000 primary sampling units (PSUs). The 2001 population census enumerator areas (EAs^1^In order to collect data for censuses, Statistics South Africa (Stats SA) demarcates work areas that are manageable for one enumerator to enumerate within limited number of days, within the local municipalities and place names. Such areas are called EAs and are the units for planning, executing and capturing of census data. This spatial set of EA boundaries is updated before each census (http://mapserver2.statssa.gov.za/geographywebsite/about.html)) were used as PSUs. These PSUs were drawn, with probability proportional to size, from a pre-census 2001 list of EAs provided by Statistics South Africa. The Master Sample focused on dwelling units or visiting points as secondary sampling units which have been defined as residential stands, addresses, structures, flats and homesteads.

The sample was stratified to include all nine provinces (Table 1) and geographic areas (formal urban, informal urban, formal rural, tribal) and ethnic groups (black, white, mixed ancestry, and Indian). The tribal areas refer to predominantly rural areas where traditionally chiefs still make decisions on matters under their jurisdiction. These areas include mostly those people living in the former TBVC homelands. TBVC refers to Transkei, Bophuthatswana, Venda and Ciskei. The age categories included 16-24; 25-34; 35-49 and 50 or more years. The Living Standards Measurement (LSM) system was used to classify people according to their living standards, using criteria such as degree of urbanization and ownership of cars and major appliances to place people in low or high categories [9].

**Table 1:** Mean dietary diversity score (DDS) and percent of adults having consumed an item from the food groups, by province

	Province									RSA
	Western Cape	Eastern Cape	Northern Cape	Free State	KwaZulu- Natal	North West	Gauteng	Mpumalanga	Limpopo	All
Sample size	441	446	228	241	630	136	613	246	306	3287
Mean DDS 95% CI ^a^	4.78 [A] 4.66 - 4.90	3.38[E][F] 3.22 - 3.54	4.05 [B][C][D] 3.85 - 4.26	4.40[B] 4.23 - 4.58	3.97 [C][D] 3.81 - 4.12	3.72 [D][E] 3.43 - 4.01	4.22 [C][B] 4.08 - 4.36	4.14 [C][B] 3.95 - 4.33	3.24 [F] 3.03 - 3.45	4.02 3.96 - 4.07
										
Percent of adults consuming at least one item from the following groups (CI^a^):										
1.Cereals/roots/tubers	99^b^	100	99	100	100	100	100	100	100	99.7
	98 - 100	99 - 100	98 - 100	99 - 100	100 - 100	100-100	100-100	99-100	99 - 100	99.5-99.9
2.Vitamin A-rich fruit and vegetables	14	16	14	26	12	21	20	22	14	17
	10 - 18	12 - 20	11 - 18	21 - 32	9 - 15	14 - 29	16 - 23	17 - 27	9 - 18	15 - 18
3.Other fruit-not vitamin A-rich	23	15	0.23	31	28	18	38	15	18	25
	18 - 27	12 - 19	18 - 29	26 - 37	24 - 32	11 - 0.25	34 - 0.43	10 - 20	14 - 22	24 - 27
4.Other vegetables- not vitamin A-rich	69	48	57	49	41	40	51	70	49	52
	64 - 74	43 - 52	49 - 65	41 - 57	35 - 47	32 - 48	47 - 56	66 - 75	41 - 57	50 - 54
5.Legumes & nuts	20	24	11	14	25	10	15	15	8	18
	16 -25	21 - 27	7 - 14	9 - 19	22 - 29	5 - 14	0.12 - 0.18	0.10 - 0.19	5 - 12	16 - 19
6.Fats & oils	66	21	40	43	39	30	28	56	26	38
	61 -70	18 - 25	33 - 47	35 - 52	34 - 45	19 - 41	24 - 0.31	48 - 65	19 - 32	36 - 40
7. Meat/poultry/fish	87	54	89	83	77	80	88	76	73	78
	83 -91	49 - 58	85 - 94	77 - 90	73 - 80	72 - 88	85 - 91	71 - 81	67 - 79	77 - 80
8.Dairy	77	44	58	72	59	55	64	42	24	56
	72 - 81	39 - 49	51 - 64	66 - 78	54 - 64	47 - 0.64	60 - 68	34 - 0.49	17 -30	55 -58
9. Eggs	24	17	14	22	16	18	18	19	13	18
	20 - 28	13 - 21	9 - 18	17 - 27	12 - 19	10 - 25	15 - 22	14 - 23	9 - 16	16 - 19

^a^CI = Confidence interval selected because of data being stratified;

^b ^% of people consumed from the cereal group

[A]; [B], [C],[D],[E],[F]: different symbols indicate which mean DDS (comparing the nine provinces) are significantly different, Bonferroni multiple comparison test, p < 0.05

In 2009, 3827 sampling units (projected sample) were randomly selected for a nutrition module included in the SASAS survey on food diversity and population consumption of all foods by the Centre for the Study of the Social and Environmental Determinants of Nutrition (CSSEDN), Knowledge Systems, HSRC. The nutrition module comprised a face validated questionnaire (evaluated by four PhDs in nutrition), which also included a non-quantified 24 hour recall. Results presented here are only for the 24 hour recalls. The questionnaire was translated into 11 official languages of South Africa and back-translated to ensure retention of meaning. Trained interviewers completed the questionnaires while interviewing the randomly selected participants. Quality of data was assured by telephonic and physical back checks on 10% of questionnaire interviews. These were undertaken to check that interviewers had visited the homes they were required to visit and secondly to check the correctness of data entered.

The realized sample comprised 3287 adults of which 76.6% were black; 10.9% white and 12% other ethnic groups. The sample was weighted to reflect the actual population distribution.

The final sample provided dietary information by means of a non-quantified 24 hour recall face validated questionnaire while being interviewed at their randomly selected households. The 24 hour recall included the interviewer documenting all foods and drinks consumed by the person during the previous 24 hours. The survey was undertaken in October/November 2009 and hence reflects consumption of foods consumed in spring and summer. A dietitian checked and cleaned the final data. Mixed dishes were coded simply by entering every food item in the dish since quantities or proportions were not required. A Dietary Diversity Score (DDS) was calculated by counting each of nine food groups. The food groups were the same as those used in an earlier validation study on children [6]. These were calculated as follows: The South African Food Composition Tables [10] were used to group food items. Each specific food item was included in a group of nine selected food groups as used in an earlier study on children [6]. Since national dietary data is not available on adults the children's mean DDS of 4 was used as a reference point [6]. A score below 4 would be indicative of poor dietary diversity (and by association poor food security) while a score of nine would represent a very varied diet. Each food group was only counted once when calculating DDS. The nine groups used were: 1) cereals/roots/tubers; 2) meat/poultry/fish; 3) dairy; 4) eggs; 5) vitamin A rich fruit and vegetables; 6) legumes; 7) other fruit; 8) other vegetables; 9) fats and oils. The results also included calculating the proportion of people who had consumed a food group at least once. Logistic regression was done using DDS as the response variable and evaluating the risk of different determinants on DDS while adjusting for the confounders, of gender and ethnicity. Chi-square analyses and the Bonferroni multiple comparison test were used to test for differences between and within groups.

The survey received ethics approval [REC 8/1/12/09/07] from the HSRCs Ethics Committee. All participants signed informed consent and were assured that all information would be treated confidentially. Participants who were 16 and 17 years old signed assent and parental/guardian consent was obtained, whereas those 18 years and older signed their own consent in accordance with the Ethics Committee of the HSRC

## Results

**Figure 1:**
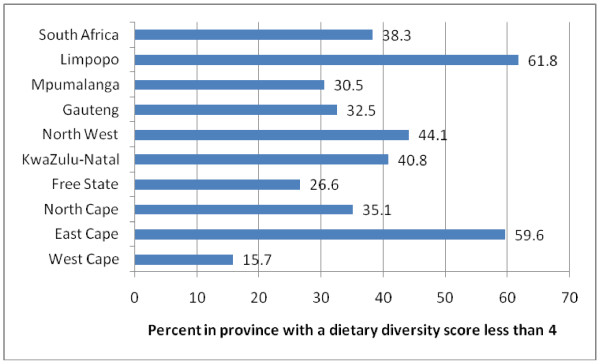
**Percentage population (with 95**^**th **^**CI) in each province having a dietary diversity score less than 4**.

At the national level the mean DDS was 4.02 [Confidence Interval (CI): 3.96 - 4.07)] and there were significant provincial differences (Table 1; Figure 1). The four provinces with the highest prevalence of poor dietary diversity (DDS <4) were the Eastern Cape (59.6%), KZN (40.8%), North West (44.1%) and Limpopo (61.8%). Only 15.7% of participants in Western Cape had a low score. The most commonly consumed food groups were cereals/roots; meat/fish; dairy and vegetables (other than vitamin A rich). Eggs, legumes, vitamin A rich fruit and vegetables were the least consumed (Table 1).

**Figure 2:**
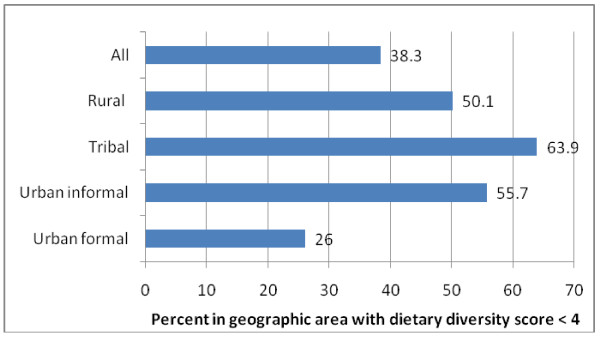
**Percent of the population (with 95**^**th **^**CI) with a dietary diversity score less than 4 according to geographic area**.

A comparison of geographic areas (Table 2) showed that urban formal areas had the highest mean DDS of 4.42[CI: 4.34-4.50] while tribal areas had the lowest mean score; which was significantly lower than any other group (p < 0.05) . Just over one third of households nationally and just under two thirds of households in tribal areas had a DDS <4, respectively (Figure 2). After cereals, in all geographic areas, meat and dairy were most commonly consumed food groups. Chi-square analysis showed significant differences between geographic areas, particularly in terms of other fruit ( not vitamin A rich) (p < 0.001); other vegetables ( not vitamin A rich) (p < 0.05); legumes and nuts (p < 0.01); fats and oils (p = < 0.001); meat (p < 0.001); dairy (p < 0.001); and eggs (p < 0.001).

**Table 2:** Mean dietary diversity score (DDS) and percent adults having consumed an item from the food groups, according to geographic area

	Geographic area				RSA
	Urban, formal	Urban, informal	Tribal	Rural,	All
Sample size	2024	309	599	355	3287
Mean DDS	4.42 [A]	3.46 [B]	3.17 [C]	3.64 [B]	4.02
95%CI^a^	4.34 - 4.50	3.30 - 3.61	3.05 - 3.29	3.46 - 3.81	3.96 - 4.07
					
Percent of adults consuming at least one item from the following groups (CI^a^):					
1.Cereals/roots/tubers	100^b^	100	100	100	99.7 9
	99 - 100	99 - 100	100 - 100	99 - 100	99.5-99.
2.Vitamin A rich fruit and vegetables	18	17	14	14	17
	16 - 20	12 - 21	12 - 17	11 - 18	15 - 18
3.Other fruit- not vitamin A-rich	33***	16	9	15	25
	31 - 36	12 - 19	7 - 11	9 - 20	24 -27
4.Other Vegetables	54*	45	51	50	52
	51 - 56	39 - 51	46 - 56	43 - 57	50 - 54
5.Legumes & nuts	16**	18	23	17	18
	14 - 18	14 - 22	20 - 26	13 - 20	16 - 19
6.Fats & oils	42***	0.29	26	42	38
	40 - 45	24 - 35	22 - 31	36 - 48	36 -40
7. Meat/poultry/fish	89***	66	55	65	78
	88 - 91	60 - 72	50 - 59	59 - 70	77 - 80
8.Dairy	70***	39	27	45	56
	67 - 72	34 - 44	24 - 31	39 - 51	55 - 58
9. Eggs	20***	17	11	17	18
	18 - 22	12 - 21	8 - 14	13 - 21	16 - 19

^a ^CI= Confidence interval selected because of data being stratified; ^b ^= 99% and 100 = 100% of people consumed from the cereal group

[A]; [B] and [C]: different symbols indicate which mean DDS values (comparing the four geographical areas) are significantly different, Bonferroni multiple comparison test, p < 0.05.

* Significant relationship between geographic group and whether or not subjects consume food from a specific group, Chi-square test, p < 0.05.

**Significant relationship between geographic group and whether or not subjects consume food from a specific group, Chi-square test, p < 0.01.

***Significant relationship between geographic group and whether or not subjects consume food from a specific group, Chi-square test, p < 0.001.

**Figure 3:**
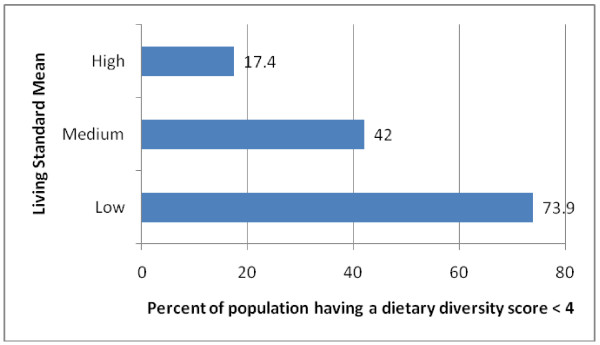
**Percent of the population (with 95**^**th **^**CI) having a dietary diversity score less than 4 according to living standards**.

There were significant differences in DDS by LSM (p < 0.05) with the lowest LSM group having the lowest DDS of 2.93 [CI:2.81-3.05] (Table 3). The highest prevalence (73.9%) of a DDS less than 4 was also recorded for the lowest LSM group (Figure 3). There were no age and gender differences in the mean DDS. In terms of food groups it should be noted that with the exception of cereals and legumes the low LSM group had significantly lower intake from all food groups while the highest LSM group had the highest in each group, excluding legumes and nuts. The discriminatory value of using DDS is further shown by the additional finding that the means of the three LSM groups differ significantly.

**Table 3:** Mean dietary diversity score (DDS) and percentage adults having consumed an item from the food groups, by Living Standard Measure, age and gender

	Living Standard Measure			Age category				Gender		RSA
	Low	Medium	3: High	16-24 years	25-34 years	35-49 years	50+ years	Male	Female	All
Sample size^c^	585	1320	1219	648	779	1001	859	1336	1951	3287
Mean DDS	2.93 [C]	3.84 [B]	4.72 [A]	3.93	3.92	4.09	4.08	4.01	4.02	4.02
(95% CI^a^)	2.81 - 3.05	3.76 - 3.93	4.64 - 4.80	3.82 - 4.05	3.82 - 4.02	3.98 - 4.19	3.98 - 4.19	3.92 - 4.09	3.95 - 4.10	3.96 - 4.07
										
Percent of adults consuming at least one item from the following groups (CI^a^):										
1.Cereals/roots/tubers	99^b^	100	100	100	100	100	100	100	100	99.7
	99 - 100	-	99 - 100	99 - 100	99 - 100	99 - 100	99 - 100	100 - 100	99 - 100	99.5-99.9
2.Vitamin A rich fruit and vegetables	14**	15	20	13**	16	20	17	15*	18	17
	11 - 16	13 - 17	17 - 22	10 - 16	13 - 18	17 - 22	15 - 20	13 - 17	16 - 20	15 - 18
3.Other fruit- not vitamin A-rich	5***	18	42	27	25	26	22	26	25	25
	3 - 8	16 - 0.20	39 -45	24 - 30	22 - 29	23 - 29	19 - 25	23 - 28	23 - 27	24 - 27
4.Other Vegetables- not vitamin A-rich	49***	46	60	46***	48	55	58	51	53	52
	44 - 54	43 - 49	57 - 63	42 - 50	44 - 52	51 - 58	55 - 62	48 - 54	50 -55	50 - 54
5.Legumes & nuts	24***	18	15	15	17	19	19	16*	19	18
	21 - 27	15 - 20	13 - 17	12 - 17	14 - 19	17 - 22	17 - 22	14 - 18	17 - 21	16 - 19
6.Fats & oils	22***	42	43	40	37	38	38	0.36	39	38
	18 - 26	38 - 45	40 - 46	36 - 43	34 - 41	35 - 41	35 - 42	33 - 39	37 - 42	36 - 40
7. Meat/poultry/fish	45***	78	93	79	81	77	76	81**	76	78
	41 - 49	75 - 80	91 - 94	76 -83	78 - 84	74 - 80	73 - 79	78 - 83	74 - 78	77 - 80
8.Dairy	25***	48	80	54***	50	58	62	57	56	56
	22 - 29	45 - 51	77 - 82	50 - 58	46 - 53	55 - 61	59 - 65	55 - 60	53 - 58	55 - 58
9. Eggs	9***	20	19	20	19	17	15	19	17	18
	7 - 11	18 - 23	17 - 21	17 - 23	16 - 22	15 - 19	13 - 18	17 - 21	15 - 18	16 - 19

^a ^CI = Confidence interval- selected because of data being stratified; ^b ^99 = 99% and 100 = 100% of people consumed from the cereal group; sample size does not always add up to n = 3287 since some questions were not answered or were unknown by participants

[A]; [B] and [C]: different symbols indicate which mean DDS values (comparing the LSM categories) are significantly different, Bonferroni multiple comparison test, p < 0.05. No significant differences between the age categories or the gender categories with regards to DDS value.

*Significant relationship between LSM, age category and gender groups respectively, and whether or not subjects consume food from a specific group, Chi-square test, p < 0.05.

**Significant relationship between LSM, age category and gender groups respectively, and whether or not subjects consume food from a specific group, Chi-square test, p < 0.01.

***Significant relationship between age category and gender groups respectively, and whether or not subjects consume food from a specific group, Chi-square test, p < 0.0001.

**Figure 4:**
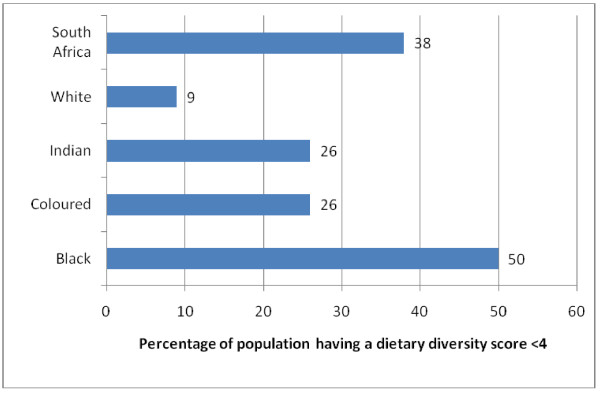
**Percentage of the population (with 95**^**th **^**CI) having a dietary diversity score less than 4 according to ethnicity**.

Differences in DDS by ethnic group (Table 4) Indicated that the black ethnic group had the lowest mean DDS of 3.63 [CI:3.55-3.71] and highest percentage of individuals with a low DDS (50%)(Figure 4) of the population with a DDS < 4, which was significantly lower than all other ethnic groups (p < 0.05). By contrast the white ethnic group had the highest mean DDS (4.96) and lowest percentage (9%) of individuals with a DDS < 4(p < 0.05).

**Table 4:** Mean dietary diversity score (DDS) and percent of adults having consumed an item from the food groups by ethnicity

	Ethnicity				RSA
	Black/African	Mixed ancestry	Indian/Asian	White	All
Sample size	1941	604	389	353	3287
Mean DDS	3.63 [C]	4.43 [B]	4.44 [B]	4.96 [A]	4.02
95% CI^a^	3.55 - 3.71	4.30 - 4.56	4.29 - 4.58	4.82 - 5.10	3.96 - 4.07
					
Proportions of adults consuming at least one item from the following groups (CI^a^):					
1.Cereals/roots/tubers	100^b^	100	100	99	99.7
	100 - 100	99 - 100	99 - 100	99 - 100	99.5-99.9
2.Vitamin A rich fruit	17**	15	12	22	17
and vegetables	16 - 19	12 - 18	8 - 16	17 - 26	15 - 18
3.Other fruit- not vitamin A-rich	20***	22	37	48	25
	18 - 21	19 - 25	33 - 42	43 - 54	24 - 27
					
4.Other vegetables- not vitamin A-rich	47***	58	49	71	52
	45 - 50	54 - 62	43 - 55	66 - 76	50 - 54
5.Legumes & nuts	19***	17	20	9	18
	17 - 20	14 - 20	16 - 24	6 - 12	16 - 19
6.Fats & oils	32*** 29 - 34	54 49 - 59	42 35 - 48	43 37 - 50	38 36 - 40
7. Meat/poultry/fish	69***	89	90	94	78
	67 - 72	86 - 92	87 - 93	92 - 97	77 - 80
8.Dairy	43***	69	80	86	56
	40 - 45	64 - 73	76 - 85	82 - 90	55 - 58
9. Eggs	17**	20	14	23	18
	15 - 18	17 - 23	10 - 17	18 - 28	16 - 19

^a ^CI= Confidence interval- selected because of data being stratified; ^b ^99 = 99% and 100 = 100% of people consumed from the cereal group

[A]; [B] and [C]: different symbols indicate which mean DDS values (comparing the four ethnic groups) are significantly different,

Bonferroni multiple comparison test, p < 0.05

**Significant relationship between ethnic groups and whether or not subjects consume food from a specific group, Chi-square test, p < 0.01.

***Significant relationship between ethnic groups and whether or not subjects consume food from a specific group, Chi-square test, p < 0.0001.

The relative odds of having a DDS < 4 compared with a DDS >= 4 (Table 5) was the highest under the following conditions: When a river was the source of drinking water (OR 7.060) the household had no toilet (OR 3.350) and no access to electricity (OR 2.310); source of income was casual work (OR 2.769) and being chronically sick or disabled (OR 2.130). A participant was most likely to buy from the small local store (spaza) in the immediate vicinity and having a limited variety of food items (OR 1.979) and to live in a traditional mud house (OR 2.394). Determinants which were protective against having an odds DDS <4 compared with a DDA >= 4 were those related to better socio-economic status, for instance earning a salary reduced the odds ratio of low DDS by nearly 48.4%(OR 0.584) water in the house by about 54.4%(OR 0.356) and electricity by nearly 61.1% (OR = 0.389).

**Table 5:** Odds ratios of household factors associated with a dietary diversity score < 4, adjusted for ethnicity and gender

	Logistic regression with question only, plus gender and ethnicity as confounders	
	**Odds Ratio**	**95% Confidence Interval**

**Employment status**		

Employed full time	0.672	0.430 - 1.050

Employed part time	1.074	0.642 - 1.797

Casual work	2.769*	1.447 - 5.298

Unemployed, not looking for work/pensioner/housewife/student	1.019	0.653 - 1.591

Unemployed/housewife looking for work	1.431	0.913 - 2.244

Disabled or chronically ill	2.130*	1.034 - 4.390

**Income**		

Receive salary/wages	0.584*	0.414 - 0.826

Remittances	1.003	0.611 - 1.646

Pensions and/or grants	1.357	0.944 - 1.950

No income	1.349	0.752 - 2.420

**Where buy most of household foods**		

Local spaza	1.979*	1.150 - 3.406

Big supermarket close by	0.573*	0.349 - 0.939

Big supermarket far away	0.944	0.575 - 1.551

**Housing**		

Formal: house or apartment	0.570	0.282 - 1.151

Traditional dwelling	2.394*	1.121 - 5.116

Townhouse	0.310*	0.140 - 0.688

Dwelling in backyard	0.706	0.324 - 1.537

Informal dwelling	1.380	0.663 - 2.870

**Water**		

Water in home	0.356*	0.265 - 0.478

Water in yard	0.694*	0.505 - 0.954

Free communal tap	1.190	0.849 - 1.669

Free from neighbor	2.275*	1.063 - 4.870

Communal borehole	1.599	0.805 - 3.173

River, stream or spring	7.060*	3.096 - 16.101

**Toilet**		

Flush toilet	0.467	0.155 - 1.406

Chemical toilet	4.226	0.987 - 18.099

Pit latrine/bucket	1.191	0.395 - 3.592

None	3.350*	1.062 - 10.562

**Electricity**		

In-house meter	0.389*	0.208 - 0.727

In-house pre-paid meter	0.611	0.330 - 1.133

Connected t other sources	1.428	0.649 - 3.142

No access	2.310*	1.198 - 4.453

**Other assets (individually assessed)**		

Refrigerator	0.274*	0.229 - 0.328

Microwave	0.279*	0.236 - 0.330

VCR	0.608*	0.495 - 0.747

TV	0.319*	0.264 - 0.385

Telephone	0.391*	0.317 - 0.483

Kitchen sink	0.263*	0.219 - 0.316

Home security	0.359*	0.278 - 0.465

Motor vehicle	0.326*	0.270 - 0.394

Mobile phone	0.473*	0.389 - 0.575

Radio	0.725*	0.622 - 0.845

Music centre	0.407*	0.347 - 0.478

DVD player	0.375*	0.320 - 0.440

## Discussion

In order to achieve *nutrition security *individuals need access not only to sufficient, safe, and nutritious food, but also to other essential factors [11]. These include: (i) access to health-care services; (i) access to safe water, hygiene, and sanitation; and (iii) knowledge about child care, food hygiene and preparation, and a healthy environment. Food security on the other hand indicates whether sufficient and adequate food is available to the individual or household, and dietary diversity is an outcome measure of this. A varied diet is associated with a number of improved health outcomes, including birth weight; child anthropometric status and improved iron status [12]. In the present study we did not try to evaluate nutrition security and hence do not know whether other factors like access to health care and safe water are adequate. In order to evaluate nutrition security these factors would also need to be evaluated. This however was not within the scope of our study. However, we recommend that these aspects also be included in future national studies investigating food security.

Data from the present study portray the situation regarding dietary variety of the South African population, and by association, food security. The findings are a cause for serious concern. Dietary variety is low overall and certainly not in line with the food-based dietary guideline promoted by the Department of Health (DOH) in South Africa which states "eat a variety of foods"[13]. Dietary variety was particularly low in the low LSM group and in black South Africans. Nearly 40% of South Africans only had between one and three different food groups on the day prior to the survey; these being a cereal, meat or chicken and a vegetable other than a vitamin A rich one. The most neglected food groups were vitamin A rich fruit and vegetables; and legumes and nuts, despite the DOH dietary guidelines which state: "Eat plenty of fruit and vegetables" and "Eat plenty of dry beans, lentils and soya regularly"[13]. It is possible that these health promotion messages may not be reaching, or be understood, by those who need them. Poor people often do not have access to a variety of food- and unless access is being addressed, knowledge on the food-based dietary guidelines will probably have little effect. Furthermore, it also needs to be realized that including more variety in the diet will in all likelihood increase the cost.

A comparison of the DDS of the present study (national mean DDS = 4.02), was done with some local studies. Firstly, a survey in Sekhukune in Limpopo, confirmed the link between food security and dietary diversity [14]. They found a significant inverse correlation between the "household food insecurity and access scale" (HFIAS) and dietary diversity (p < 0.01). A study on the low income elderly in Sharpeville, South Africa showed a DDS of 3.41(SD 1.34), similar to that of the low LSM in the present study [15].They also determined that having a better DDS results in a better mean nutrient adequacy ratio. Another study in South Africa determined the DDS of infants (6-24 months) [16]. They found that low dietary diversity was more common in HIV infected children than those who were not ( crude odds ratio (OR), 2.59; 95% CI, 1.52 to 4.41).

In terms of studies in other developing countries who have evaluated DDS, a mean DDS of 4.91 was found in Filipino children aged 24-71 months using a score out of nine groups [17]. In Burkino Faso it was 4.6, in Laos 5.2 and 3.3 in Northern Uganda [18]. It is apparent that poor dietary variety is a feature of many developing countries, and is not restricted to the South African population.

Results from the present national survey indicate that environmental factors are important determinants associated with household food security. However, it is also important to realize that improving the environment is not necessarily going to lead to better household food security if people do not have access to food. Furthermore, it is known that *nutrition security *cannot be achieved without food security, knowledge and skills to improve dietary intake and access to health services [11]. Using DDS as a proxy for food security showed that for example, a river water source was associated with a seven fold relative odds ratio of having a DDS < 4 compared with those with a DDS >= 4. Other factors which contributed to low DDS, included: having no toilet; living in a traditional type house; and no access to electricity. Health care was also regarded as a contributing factor to nutrition security since those chronically ill (by implication many HIV) or disabled had twice the odds of having a low DDS. Indeed, the majority of environmental risk factors are the direct outcome of poverty and are inter-related.

A recent publication indicates that a limited understanding of the current situation of a population/community is likely to hamper the development of effective strategies to improve the nutrition situation [19]. In this regard, the determinants of poor food security are multiple and complex comprising both environmental and social factors, with poverty being one of the primary contributors [20]. Food-based approaches are most likely to be successful, if they are part of a long term process which leads to economic growth.

One approach, based on the current findings, would be to identify those communities which use a river or stream as a source of drinking water. Such communities should be given priority regarding the piping of water to their homes and in further development initiatives. Within communities, families without a toilet in their home or yard and no access to electricity can be targeted for individual social support. The data from this survey do imply that improvements to the environment (housing, water, electrification, sanitation) will lead to improved *nutrition *security. However, to improve food security will still require better access to food. These are hence aspects that policymakers need to focus on, in addition to direct nutrition strategies which improve access to food.

In terms of access to food it should be noted that buying food from local spaza shops also doubled the relative odds of having a poor DDS while having a big supermarket close was protective against a DDS < 4. Spaza shops generally sell very basic necessities and generally are not good sources of fresh fruit and vegetables. Furthermore, studies in South Africa have shown that food prices are highest where the poor live [21,22]. Hence, availability and access to healthy foods needs to be improved for those residing far from large supermarkets. The issue of small-scale agricultural production is one that needs to be continuously evaluated despite lack of access to land by the large majority of people. Government and the private sector need to be creative in this regard and should focus on ways to make access to land for food production possible [23].

At this point in time it is appropriate to question what we still need to know about the determinants of poor dietary variety in the South African population. A search of the literature reveals that there is a paucity of data on people's knowledge and practices with regard to the food-based dietary guidelines promoted by the DOH. In this regard, one needs to know how effective the marketing of the dietary guidelines are, and whether people interpret and practice them effectively. Furthermore, one needs to know whether having better nutrition knowledge will impact favorably on households which are food insecure due to lack of access to food. Knowledge does not necessarily imply access to food. In this regard, Hart (2009)[24] emphasized the essential importance of more qualitative and in-depth studies of household food insecurity to determine why some households remain more or less constantly food insecure while some in the same community do not.

There is a large evidence base for school curriculum-based nutrition education programs in terms of improved knowledge, self-efficacy and attitudes leading to improved nutritional behavior [25]. Additionally, if this is coupled with healthy foods with plenty of variety in the primary school nutrition program, one would be able to provide both knowledge and access to food. It is also essential that school policies reinforce healthy eating behavior in terms of the type of foods sold at the school. The introduction of the food-based dietary guidelines into the Life Orientation (LO) curriculum at schools is currently being evaluated at 8 schools in the Western Cape Province [26,27]. The LO curriculum covers a range of life skills aimed at promoting healthy and responsible citizens. One way in which one could improve intake of vitamin A rich fruits and vegetables would be to have a school vegetable garden and teach children how to grow their own vegetables.

Lastly, it needs to be recognized that nutrition security of individuals and households are influenced by a myriad of factors, particularly those related to the immediate environment. Ideally, South Africa should strive for all households to have access to food, water, sanitation and health care. This can only happen if economic growth takes place and there are employment opportunities for all.

## Conclusions

Overall the majority of South Africans consumed a diet low in dietary variety. The tribal areas and informal urban areas were worst affected and eggs, legumes and vitamin A rich fruit and vegetables, were the least consumed.

## Competing interests

This study was funded by the Human Sciences Research Council and there is no conflict of interest.

## Authors' contributions

DL and NPS planned and wrote the article. JN was responsible for data analyses and interpretation thereof.
